# Non-O1, non-O139 *Vibrio cholerae* bacteraemia: case report and literature review

**DOI:** 10.1186/s40064-015-1346-3

**Published:** 2015-10-05

**Authors:** S. Deshayes, C. Daurel, V. Cattoir, J.-J. Parienti, M.-L. Quilici, A. de La Blanchardière

**Affiliations:** Service des Maladies Infectieuses et Tropicales, CHU Côte de Nacre, avenue Côte de Nacre, 14033 Caen Cedex 9, France; Service de Microbiologie, CHU Côte de Nacre, avenue Côte de Nacre, 14033 Caen Cedex 9, France; Unité de Biostatistiques, CHU Côte de Nacre, avenue Côte de Nacre, 14033 Caen Cedex 9, France; Centre National de Référence des Vibrions et du Choléra, Institut Pasteur, 28 rue du Docteur Roux, 75724 Paris Cedex 15, France

**Keywords:** Non-O1 *Vibrio cholerae*, Bacteraemia, Abscess

## Abstract

Non-O1, non-O139 *Vibrio cholerae* (NOVC) are increasingly frequently observed ubiquitous microorganisms occasionally responsible for intestinal and extra-intestinal infections. Most cases involve self-limiting gastroenteritis or ear and wound infections in immunocompetent patients. Bacteraemia, which have been described in patients with predisposing factors, are rare and poorly known, both on the clinical and therapeutic aspects. We describe a case of NOVC bacteraemia and a systematic literature review in PubMed conducted up to November 2014 using a combination of the following search terms: “*Vibrio cholerae* non-O1” and “bacter(a)emia”. The case was a 70 year-old healthy male subject returning from Senegal and suffering from NOVC bacteraemia associated with liver abscesses. Disease evolution was favourable after 2 months’ therapy (ceftriaxone then ciprofloxacin). Three hundred and fifty cases of NOVC bacteraemia have been identified in the literature. The majority of patients were male (77 %), with a median age of 56 years and presenting with predisposing conditions (96 %), such as cirrhosis (55 %) or malignant disease (20 %). Diarrhoea was inconstant (42 %). Mortality was 33 %. The source of infection, identified in only 25 % of cases, was seafood consumption (54 %) or contaminated water (30 %). Practitioners should be aware of these infections, in order to warn patients with predisposing conditions, on the risk of ingesting raw or undercooked seafood or bathing in potentially infected waters.

**Electronic supplementary material:**

The online version of this article (doi:10.1186/s40064-015-1346-3) contains supplementary material, which is available to authorized users.

## Background

The genus *Vibrio* belongs to the *Vibrionaceae* family. *Vibrio* species are halophilic facultative anaerobic Gram-negative bacilli, which are ubiquitously distributed in marine and estuarine environments. Their presence is particularly well documented in Asia and Latin America and in the coastal waters of the Gulf of Mexico. Their density is increasing, particularly in filter-feeding shellfish, associated with high surface water temperature, especially during warmer months (13–25 °C), secondary to the proliferation of phytoplankton and zooplankton (Crim et al. 2014; Harris et al. 2012; Huehn et al. 2014). There is an increasing trend towards infection due to *Vibrio*. Despite under-diagnosis and under-reporting, especially for milder cases, they are the 6th pathogen transmitted through food in the USA, after *Salmonella*, *Campylobacter*, *Shigella*, *Cryptosporidium* and Shiga toxin-producing *Escherichia coli* (Crim et al. 2014; Huehn et al. 2014).

Over 200 serogroups compose the *V. cholerae* species, based on the surface O antigen of the lipopolysaccharide (Harris et al. 2012). The two major serogroups, O1 and O139, are responsible for epidemic cholera, an acute diarrheal disease leading to 28,000–142,000 deaths every year, according to the WHO. Bacteraemia associated with choleragenic vibrios is rare, possibly thanks to the ability of the cholera toxin, a non-invasive enterotoxin, to suppress induction of inflammation during infection (Fullner et al. 2002).

Non-choleragenic vibrios, including the other serogroups of the *V. cholerae* species, and other species of *Vibrio*, mainly *V. alginolyticus*, *V parahaemolyticus* and *V. vulnificus*, can lead to intestinal infections (gastroenteritis) as well as extra-intestinal manifestations (wound infections, external otitis and bacteraemia) through invasive mechanisms, with significant mortality.

In recent years, there has been an increase in the number of reports of infections involving non-O1, non-O139 *V. cholerae* (NOVC). The majority were case reports of self-limiting gastroenteritis, ear and wound infections in immunocompetent patients or bacteraemia in immunocompromised hosts with predisposing medical conditions (Petsaris et al. 2010).

However, NOVC infection may rarely lead to invasive extraintestinal infection and potentially fatal bacteraemia in healthy patients (Mannion and Mellor 1986). We report a case of NOVC bacteraemia with liver abscesses in a French immunocompetent male subject returning from Senegal, and discuss the epidemiology, the clinical manifestations, the predisposing factors and the antimicrobial therapy of NOVC bacteraemia through a review of 350 identified cases.

## Methods

A review of the literature in English, French and Spanish was conducted via an electronic search on MEDLINE by crossing the key words “*Vibrio cholerae* non-O1” and “bacter(a)emia”. We also retrieved the articles in the reference lists of papers found in our searches. The literature search period ranged from the first described case in 1974 to November 2014.

Statistical analysis was performed using R 3.0.3 statistical software. Categorical variables were reported as percentages and compared using Chi square or Fisher’s exact tests according to expected frequencies. Continuous variables were expressed as means and analysed using Student’s t-test. A p-value <0.05 was considered to be statistically significant.

## Case report

A 70 year-old man was referred to the Infectious Diseases Unit in our institution in April 2010 for fever and watery diarrhoea, after spending 3 weeks in Senegal.

The patient presented with a previous history of myocardial infarction, hypertension, hepatitis A in 1954 and cholecystectomy. No alcohol abuse, malignant or immunocompromising disease was reported.

The patient presented with a single episode of watery diarrhoea, vomiting and dizziness associated with a short loss of consciousness on the day of his return to France and a 3-kg weight loss. Over the following days, he complained of high fever with chills and abdominal pain. The patient stated no history of bathing in the sea or in fresh water; however, he reported important consumption of fish and shellfish, sometimes undercooked, whereas no other case was reported among his fellow travellers.

On arrival, his body temperature was 38.1 °C and his vital signs were stable. The results of physical examination were normal with the exception of abdominal tenderness, mainly on the upper right quadrant. No jaundice was reported.

**Fig. 1:**
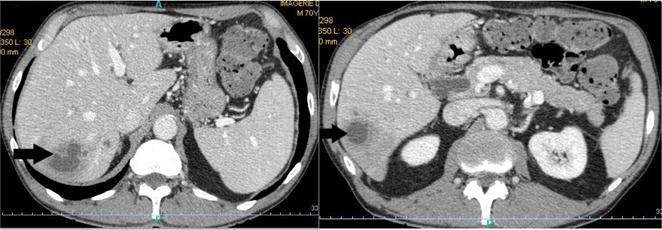
Abdominal CT showing two low density lesions in the right liver (a*rrows*), compatible with the diagnosis of liver abscesses

**Fig. 2:**
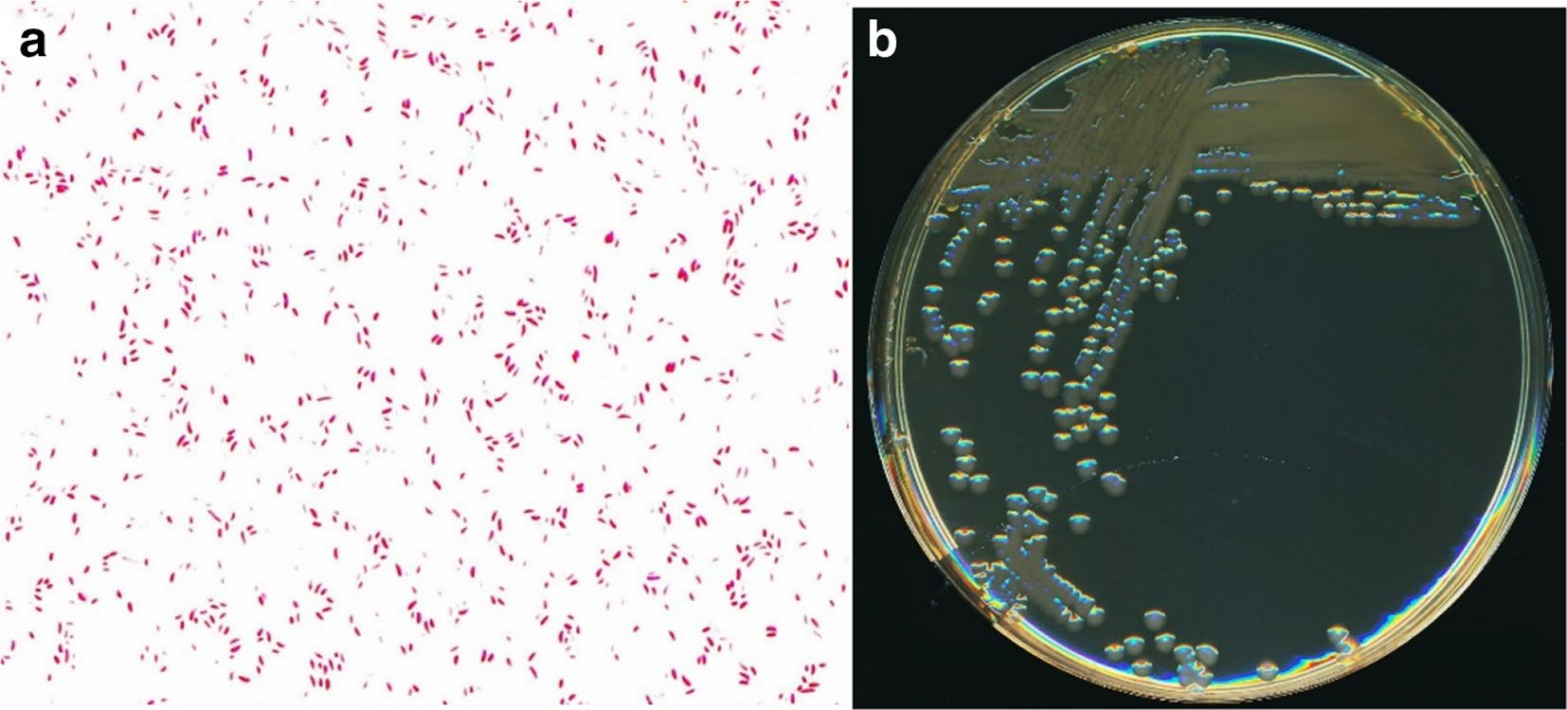
**a** Gram stain (magnificence ×1000) and **b** colonial morphology of non-O1, non-O139 *V. cholerae* grown on Trypticase-Soy agar after 18 h of aerobic incubation at 35 °C (Photos M. Auzou)

Laboratory tests revealed an increased white blood cell count (13 × 10^9^/L) and elevated C-reactive protein (397 mg/L). Serum creatinine was within the reference range. Liver function test results were elevated, including aspartate aminotransferase, 119 IU/L; alanine aminotransferase, 216 IU/L; and alkaline phosphatase, 163 IU/L, without hepatocellular insufficiency. Abdominal ultrasonography revealed two heterogenous collections from 3 to 5 cm in the right liver compatible with abscesses, confirmed by CT scan (Fig. 1). Neither of the two imaging techniques showed any signs of underlying chronic hepatopathy, nor damage on biliary ducts or portal vessel. One of the two sets of blood cultures collected upon admission yielded a Gram-negative rod, compatible with *V. cholerae* (Fig. 2). Stool cultures were negative. The strain was sent to the French National Reference Center for Vibrios and Cholera (CNRVC, Institut Pasteur, Paris, France) for confirmation of the identification by biochemical, molecular and cultural methods, agglutination with O1 and O139 antisera and search for virulence factors. The strain did not agglutinate when tested against O1 or O139 antisera. PCR techniques demonstrated the absence of the major virulence-encoding genes of toxigenic *V. cholerae*, the cholera-toxin (*ctxA* and *ctxB*) and the toxin-coregulated pilus (*tcpA*) virulence genes, and of the *stn* gene, encoding a heat-stable enterotoxin reported to contribute to the pathogenicity of NOVC. PCR was positive for the El-Tor *hlyA* gene. The bacteria was sensitive to amoxicillin, cefotaxime, ofloxacin, gentamicin, cotrimoxazole.

Empirical parenteral treatment with intravenous ceftriaxone (1 g every 24 h) was initiated then shifted to oral ciprofloxacin (500 mg every 12 h) after 15 days. Clinical evolution was favourable, with a rapid decrease in fever and resolution of abdominal pain. After 2 months’ treatment, abdominal ultrasound did not reveal any residual collection and antibiotic therapy was stopped.

## Results of the literature review

One hundred and twenty-eight articles described 350 cases of NOVC bacteraemia involving 347 patients, 3 of whom presented with a second episode (Additional file 1: Table S1) (Petsaris et al. 2010; Mannion and Mellor 1986; Lai et al. 2012; Morris et al. 1981; Anderson et al. 2004; Pierce et al. 2000; Hlady and Klontz 1996; Magnusson and Pegg 1996; Robins-Browne et al. 1977; Eltahawy et al. 2004; Marcenac et al. 1991; Ferreira et al. 2012; Zarate et al. 2011; Goei and Karthigasu 1978; Trubiano et al. 2014; Heath et al. 2001; Guard et al. 1980; Hsu et al. 2013; Huhulescu et al. 2007; Halabi et al. 1997; Berghmans et al. 2002; Kadkhoda et al. 2012; Burns et al. 1989; Ramsingh 1998; Briceno et al. 2009; Young et al. 1991; Lu et al. 2014; Farmachidi et al. 2003; Chong et al. 1985; Choi et al. 2003; Dalsgaard et al. 2000; Marek et al. 2013; Aguinaga et al. 2009; Forné et al. 1987; Prats et al. 1975; Lopez-Brea et al. 1985; Mirelis et al. 1987; Royo et al. 1993; Mauri et al. 1987; Esparcia et al. 2000; Fernández et al. 2000; Lantero et al. 1984; Folgueira et al. 1991; Fernández-Monrás et al. 1990; Catalá Barceló MT 1998; Fernández-Natal and Alcoba-Leza 1996; Calduch Broseta JV 2003; Rabadan and Vilalta 1989; Rubin et al. 1981; Nedunchezian et al. 1994; Pitrak and Gindorf 1989; Bonner et al. 1983; Newman et al. 1993; Namdari et al. 2000; Patel et al. 2009; Wagner et al. 1995; Siegel and Rogers 1982; McCleskey et al. 1986; Florman et al. 1990; West et al. 1998; Klontz 1990; Hughes et al. 1978; Restrepo et al. 2006; Safrin et al. 1988; Fearrington et al. 1974; Shannon and Kimbrough 2006; Platia and Vosti 1980; Kontoyiannis et al. 1995; Shelton et al. 1993; Crump et al. 2003; Naidu et al. 1993; Morgan et al. 1985; Lukinmaa et al. 2006; Blanche and Sicard 1994; Moinard et al. 1989; Laudat et al. 1997; Raultin and de La Roy, 1981; Couzigou et al. 2007; Issack et al. 2008; Kerketta et al. 2002; Thomas et al. 1996; Lalitha et al. 1986; George et al. 2013; Raju et al. 1990; Khan et al. 2013; Toeg et al. 1990; Rudensky et al. 1993; Farina et al. 1999; Piersimoni et al. 1991; Ismail et al. 2001; Dhar et al. 1989, 2004; Phetsouvanh et al. 2008; Feghali and Adib 2011; Tan et al. 1994; Deris and Leow 2009; Whittaker 2013; Stypulkowska-Misiurewicz et al. 2006; Albuquerque et al. 2013; El-Hiday and Khan 2006; Khan et al. 2007; Strumbelj et al. 2005; Wiström 1989; Lin et al. 1996; Ko et al. 1998; Lee et al. 1993; Chang-Chien 2006; Tsai and Huang 2009; Chan et al. 1994; Yang et al. 2008; Cheng et al. 2004; Tsai et al. 2004; Wang et al. 1991; Laosombat et al. 1996; Punpanich et al. 2011; Thisyakorn and Reinprayoon 1990; Luxsameesathaporn et al. 2012; Suankratay et al. 2001; Wiwatworapan and Insiripong 2008; Boukadida et al. 1993; Lan et al. 2014; Geneste et al. 1995; Yang et al. 2011; Thomas et al. 2007; Ou et al. 2003; Lee et al. 2007; Thamlikitkul 1990; Jesudason et al. 1991). The majority of articles were case reports, the largest series including 69 cases of bacteraemia (Ou et al. 2003). The first case was described in the USA in 1974 (Fearrington et al. 1974). One hundred and fifty-six cases (45 %) originated from Taiwan, 60/350 (20 %) from the USA and 21/350 (6 %) from Spain. Although NOVC strains are frequently found in coastal waters, only two cases have been reported in Africa. Two possible explanations are under-diagnosis due to lack of resources, and the non reporting of clinical cases. Including our own case report, 12 cases of NOVC bacteraemia have been published in France, in summer or autumn, including four imported cases from Tunisia (2), Morocco (1) and Senegal (1) (Farmachidi et al. 2003; Blanche and Sicard 1994; Moinard et al. 1989; Laudat et al. 1997; Raultin and de La Roy, 1981; Couzigou et al. 2007).

NOVC infection predominantly affected middle-aged male subjects (median age 56 years, sex-ratio 3.3) and rarely children <18 years (4.6 %). The main risk factor for NOVC bacteraemia was cirrhosis (54 %). Other risk factors were cancer or malignant blood diseases, alcoholism, other liver diseases, diabetes, and iatrogenesis (Additional file 1: Table S1).

When specified, the source of NOVC bacteraemia was most often seafood consumption (53.9 %) including oysters (9/22, 41 %), fish (5/22, 23 %), shrimps (4/22, 18 %), clams (2/22, 9 %), mussels (1/22, 4 %) and apple snail (1/22, 4 %) (Additional file 1: Table S1) (Crim et al. 2014; Morris et al. 1981; Anderson et al. 2004; Pierce et al. 2000; Trubiano et al. 2014; Halabi et al. 1997; Dalsgaard et al. 2000; Marek et al. 2013).

The clinical presentation of bacteraemia was most often hypo or hyperthermia, diarrhoea and abdominal pain. Jaundice and ascites were probably linked to cirrhosis (Additional file 1: Table S1). When specified, diarrhoea was most often watery (20/25, 80 %), rarely bloody (12 %) or with mucous (8 %). Including our patient, five hepatic abscesses were described, one of which yielded sterile blood cultures (Guard et al. 1980; Farmachidi et al. 2003; Strumbelj et al. 2005; Lai et al. 2011). Two cases of pyomyositis were also reported (Nedunchezian et al. 1994; Couzigou et al. 2007), as well as one prostatic abscess (Safrin et al. 1988), one cerebral abscess (Morgan et al. 1985) and one peritoneal abscess (Stypulkowska-Misiurewicz et al. 2006). This significant frequency of abscess, almost 5 %, had not been reported to date.

One-third of patients with NOVC bacteraemia died.

Prognostic factors were studied based on articles for which clinical outcomes were known. Hypotension and confusion or coma were statistically associated with a higher mortality, whereas digestive surgery was associated with better outcome (Additional file 1: Table S2).

## Discussion

This work represents the largest literature review on epidemiology, risk factors and prognosis of an unusual and potentially emerging pathogen, namely, non-O1, non-O139 *V. cholerae.*

The three main clinical presentations of NOVC infection are gastroenteritis, wound and ear infections and bacteraemia, the latter being the least frequent (Petsaris et al. 2010). However, strains have been isolated from various other sites, such as respiratory tract, bile, uterus, urine and cerebrospinal fluid (Lai et al. 2012). Gastroenteritis can be mild to severe, with watery more often than bloody stools, but, in all cases, prognosis is favourable (Morris et al. 1981; Anderson et al. 2004). They are however under-diagnosed, partly due to the failure of both clinicians and microbiologist to suspect vibrios as etiological agents of diarrhoea, and to the fact that many laboratories do not use the appropriate enrichment and culture media, such as thiosulfate-citrate-bile salt-sucrose (TCBS) agar, to isolate these organisms (Pierce et al. 2000). Between 1 and 3.4 % of cases of acute diarrhoea are believed to be due to NOVC, in developing and developed countries alike (Luo et al. 2013). NOVC grows in routine blood culture media. However, due to its rarity, NOVC bacteraemia is relatively unknown [17 % of NOVC infections in Florida were bacteraemia (Hlady and Klontz 1996)].

Most bacteraemia cases are associated with exposure to aquatic environments or seafood consumption, with 5.6 % of seafood samples tested in Italy positive for NOVC (Ottaviani et al. 2009), and more than one-third of seafood samples tested in Germany (Huehn et al. 2014; Cheasty et al. 1999). Bacteria may shift from the intestine to the blood through the portal vein or intestinal lymphatic system (Bonner et al. 1983). However, in almost 75 % of cases, no exposure to aquatic environments or seafood consumption was reported, suggesting other infection routes (Additional file 1: Table S1). Indeed, NOVC strains have been isolated from wild and domestic animals (Cheasty et al. 1999), while asymptomatic human carriage has also been described and two outbreaks of NOVC gastroenteritis have been linked to the consumption of grated eggs and potatoes (Morris et al. 1981; Dhar et al. 2004). NOVC can grow in water with low salinity, such as alkaline lakes, artificial waterways and sewers. It has been documented in French coastal waters (Hervio-Heath et al. 2002).

Subtyping methods, such as Pulsed Field Gel electrophoresis analysis, indicated that NOVC strains showed considerable diversity. The mechanisms underlying their virulence and in particular their capacity to invade the bloodstream are still not fully understood. These strains normally lack most of the major virulence-encoding regions of toxigenic *V. cholerae* (such as cholera toxin or toxin-coregulated pilus), but their pathogenicity has been associated with other virulence factors. Among them, a type III secretion system has been demonstrated to be involved in colonization (Chaand et al. 2015), a heat-stable enterotoxin (ST), encoded by the *stn* gene, was reported to contribute to the pathogenicity of these strains in case of gastroenteritis (Morris et al. 1990), a haemagglutinin protease (HA/P), and a haemolysin, present in *V. cholerae* O1, was suggested to be involved in the enteroinvasiveness of some NOVC isolates (Namdari et al. 2000; Luo et al. 2013; Ottaviani et al. 2009; Awasthi et al. 2013; Schirmeister et al. 2014). However, the lack of detection of *stn* gene in most of the strains associated with gastroenteritis (data from the CNRVC), the presence and expression of *hlyA* genes in strains isolated from patients without extraintestinal infection (Ottaviani et al. 2009, and data from the CNRVC) and its widespread occurrence among environmental strains, suggest that there are additional virulence factors.

Occurrence of NOVC bacteraemia is dependent on the infecting strain, but also on the health and immune status of the host. The main risk factor of NOVC bacteraemia is cirrhosis (54 %). Cirrhotic patient susceptibility to NOVC bacteraemia is thought to be linked to inflammation and oedema of intestinal mucosa with increased intestinal permeability, by-pass of the hepatic reticuloendothelial system by portal hypertension, weak opsonic activity of ascetic fluid, impairment of phagocytosis, complement deficiencies, alteration of iron metabolism and/or inhibition of chemotaxis, the precise role of each defence mechanism defect requiring further study (Anderson et al. 2004; Bonner et al. 1983; Couzigou et al. 2007; Ko et al. 1998).

In published cases of NOVC bacteraemia, there is extreme heterogeneity in antimicrobial therapy (in terms of the nature of antimicrobial agent(s), their dosage and treatment duration). In cholera, antimicrobial therapy, although adjunctive, is relatively well codified, reducing total stool volume by 50 %, the duration of shedding of viable organisms in stools from several days to 1–2 days and the quantity of rehydration fluids by 40 %. Tetracycline and azithromycin appear to be first-choice antibiotics (Leibovici-Weissman et al. 2014). Because NOVC bacteraemia is rare, no large-scale trials have been conducted. While spontaneous recovery is the rule in NOVC gastroenteritis, antimicrobial therapy is recommended in complicated forms and/or in immunocompromised patients, with a dual-agent therapy in NOVC bacteraemia according to certain authors (Couzigou et al. 2007). Tetracyclines are widely used, by analogy with cholera and because they inhibit protein synthesis, which may decrease the production of toxins (Leibovici-Weissman et al. 2014). Ko et al. (1998) reported the synergistic effect, both in vitro and in mice, of cefotaxime plus minocycline in *V. vulnificus* infections. Thus, the association of third-generation cephalosporins with a tetracycline or fluoroquinolones may offer an interesting alternative in the treatment of NOVC bacteraemia, depending on local antibiotic susceptibility testing, although recommendations regarding the choice of therapy are not conclusive. Furthermore, several cases of antimicrobial resistance have been described in environmental as well as in clinical strains, involving cefotaxime, nalidixic acid, tetracyclines, cotrimoxazole, ciprofloxacin and depending on location, certain multidrug resistant strains having been reported, particularly in India (Lu et al. 2014; Luo et al. 2013; Jagadeeshan et al. 2009). The duration of treatment is also a matter of debate, ranging from 3 to 75 days with a median of 14 days (Additional file 1: Table S1). This duration should probably be adapted according to the patient’s background, clinical presentation and severity (such as meningitis and abscess).

In our review, we didn’t observe a higher risk of mortality in patients with cirrhosis, neoplasia and iatrogenesis, unlike Ou et al. (2003). Unsurprisingly, the onset of circulatory or neurological failure was statistically associated with higher mortality. Digestive surgery seems paradoxically protective, because it does not impair the immune system, as do cirrhosis or cancer. The high mortality of bacteremia NOVC is probably due to delayed diagnosis, inadequate antimicrobial therapy and/or too short therapy duration.

## Conclusions

Ongoing global warming, anthropisation of coastal environments, international seafood trade, consumption of undercooked seafood and increase in individuals at risk will undoubtedly increase NOVC infections, especially in summer, as already demonstrated in the Baltic Sea (Huehn et al. 2014; Schirmeister et al. 2014), and will render NOVC infection an under-diagnosed, life-threatening, emerging infectious disease, involving economic issues (seafood importation) (Robert-Pillot et al. 2014). NOVC strains have been confirmed as potential contaminants of widely consumed food types in France, and are also present in shellfish and water samples collected from French coastal and estuarine areas (Hervio-Heath et al. 2002).

So there is a need to increase the capacity to ensure prompt diagnosis and public health notification and investigation for effective patient management and infection control. Physicians in temperate countries should be aware of these infections, to ensure they request the detection of *Vibrio* in faeces in cases of gastroenteritis after seafood consumption, and to ensure they warn individuals, particularly those presenting with predisposing conditions for bacteraemia (liver disease, alcoholism, diabetes, neoplasia) on the risk of ingesting raw or undercooked seafood or bathing in potentially infected waters during warm summers. All cases must be reported and confirmed by the National Reference Centre.

## Additional file

10.1186/s40064-015-1346-3 Results from systematic literature review of 350 non-O1, non-O139 *Vibrio cholerae* bacteraemia.
