# Supplementary material for: Non-O1, non-O139 Vibrio cholerae bacteraemia: case report and literature review
Source: Springerplus. 2015 Oct 5;4:575. doi: 10.1186/s40064-015-1346-3 (PMC4627963; doi:10.1186/s40064-015-1346-3)
Supplement: Supplementary file 1 — 10.1186/s40064-015-1346-3 Results from systematic literature review of 350 non-O1, non-O139 Vibrio cholerae bacteraemia. [file 40064_2015_1346_MOESM1_ESM.docx]

Table 1. Demographic and clinical parameters of 350 bacteraemia from the English, Spanish and French literature

|  | Results for the 350 bacteraemia (the denominator changes according to data availability for each case) | |
| --- | --- | --- |
| Patients | 347 / 350 | |
| Sex ratio (Male/Female) | 3.3 / 218 | |
| Median age (years) [range] | 56 / 182 [60 hours - 88 years] | |
| Predisposing factors § | Cirrhosis (%) [95% CI] | 122 / 225 (54.2) [47.7-60.7] |
|  | Other liver diseases (%) [95% CI] | 34 / 225 (15.1) [10.4-19.8] |
|  | Alcoholism (%) [95% CI] | 36 / 225 (16.0) [11.2-20.8] |
|  | Diabetes (%) [95% CI] | 31 / 225 (13.8) [9.3-18.3] |
|  | Malignant blood disease (%) [95% CI] | 29 / 225 (12.9) [8.5-17.3] |
|  | Iatrogenesis ¶ (%) [95% CI] | 27 / 225 (12.0) [7.8-16.2] |
|  | Digestive surgery (%) [95% CI] | 21 / 225 (9.3) [5.5-13.1] |
|  | Neoplasia (%) [95% CI] | 19 / 225 (8.4) [4.8-12.1] |
|  | Infants (%) [95% CI] | 7 / 225 (3.1) [0.8-5.4] |
|  | Nephropathy (%) [95% CI] | 5 / 225 (2.2) [0.3-4.1] |
|  | Malnutrition (%) [95% CI] | 4 / 225 (1.8) [0.1-3.5] |
|  | HIV (%) [95% CI] | 3 / 225 (1.3) [0-2.8] |
|  | Miscellaneous ¤ (%) [95% CI] | 6 / 225 (2.7) [0.6-4.8] |
|  | None found (%) [95% CI] | 9 / 225 (4.0) [1.4-6.6] |
| Mode of transmission § | Seafood consumption (%) [95% CI] | 48 / 89 (53.9) [43.6-64.3] |
|  | Contact with contaminated water (%) [95% CI] | 27 / 89 (30.3) [20.8-39.9] |
|  | Drinking contaminated water (%) [95% CI] | 11 / 89 (12.4) [5.5-19.2] |
|  | Wound (%) [95% CI] | 7 / 89 (7.9) [2.3-13.5] |
|  | Not found or not mentioned (%) [95% CI] | 261 / 350 (74.6) [70-79.1] |
| Clinical presentation § | Hypo or hyperthermia (%) [95% CI] | 172 / 226 (76.1) [70.5-81.7] |
|  | Diarrhoea (%) [95% CI] | 94 / 226 (41.6) [35.2-48] |
|  | Abdominal pain (%) [95% CI] | 89 / 226 (39.4) [33-45.8] |
|  | Chills (%) [95% CI] | 55 / 226 (24.3) [18.7-29.9] |
|  | Hypotension (%) [95% CI] | 50 / 226 (22.1) [16.7-27.5] |
|  | Jaundice (%) [95% CI] | 50 / 226 (22.1) [16.7-27.5] |
|  | Ascites (%) [95% CI] | 46 / 226 (20.4) [15.1-25.6] |
|  | Cellulitis (%) [95% CI] | 41 / 226 (18.1) [13.1-23.2] |
|  | Nausea or vomiting (%) [95% CI] | 36 / 226 (15.9) [11.2-20.7] |
|  | Confusion or coma (%) [95% CI] | 31 / 226 (13.7) [9.2-18.2] |
|  | Peritonitis (%) [95% CI] | 23 / 226 (10.2) [6.2-14.1] |
|  | Meningitis and/or encephalitis (%) [95% CI] | 5 / 226 (2.2) [0.3-4.1] |
| Biology | Median white blood cell count* (/mm^3^) [range] | 10000 / 79 [100-250 000 due to chronic leukemia] |
|  | Median CRP rates (mg/l) [range] | 69 / 19 [5-980] |
| Clinical outcome | Median duration of antibiotherapy (days) [range] | 14 / 74 [3-75] |
|  | Death (%) [95% CI] | 77 / 232 (33.2) [27.1-39.2] |
|  | Median time to death (days) [range] | 3.5 / 36 [1 hour-90 days] |

* Under 12 years excluded

¶ ie. corticosteroids, antacids, chemotherapy, splenectomy

¤ ie. drowning, burns, intravenous drug abuse, systemic lupus, liver transplantation, heart-lung machine

§ Total count may exceed 100% (one patient may have multiple occurrences)

Table 2. Prognostic factors in non-O1, non-O139 *Vibrio cholerae* bacteraemia

|  |  |  | Outcome | | p |
| --- | --- | --- | --- | --- | --- |
|  |  |  | Death | Favourable |  |
| Gender | Female (%) |  | 16 (37) | 27 (63) | 0.76 |
|  | Male (%) |  | 47 (35) | 89 (65) |  |
| Mean age (years) (±SD, n) |  |  | 52.63 (±19.62, 62) | 52.66 (±20.55, 115) | 1 |
| Predisposing factors | Cirrhosis (%) | Presence | 36 (40.4) | 53 (59.6) | 0.15 |
|  |  | Absence | 26 (29.9) | 61 (70.1) |  |
|  | Other liver diseases (%) | Presence | 11 (39.3) | 17 (60.7) | 0.63 |
|  |  | Absence | 51 (34.5) | 97 (65.5) |  |
|  | Alcoholism (%) | Presence | 13 (36.1) | 23 (63.9) | 0.91 |
|  |  | Absence | 49 (35) | 91 (65) |  |
|  | Diabetes (%) | Presence | 10 (37) | 17 (63) | 0.84 |
|  |  | Absence | 52 (34.9) | 97 (65.1) |  |
|  | Malignant blood disease (%) | Presence | 10 (40.4) | 15 (59.6) | 0.59 |
|  |  | Absence | 52 (34.4) | 99 (65.6) |  |
|  | Iatrogenesis (%) | Presence | 9 (37.5) | 15 (62.5) | 0.81 |
|  |  | Absence | 53 (34.9) | 99 (65.1) |  |
|  | Digestive surgery (%) | Presence | 1 (5.6) | 17 (94.4) | < 0.01 |
|  |  | Absence | 61 (38.6) | 97 (61.4) |  |
|  | Neoplasia (%) | Presence | 6 (37.5) | 10 (62.5) | 0.85 |
|  |  | Absence | 56 (35) | 104 (65) |  |
|  | Infants (%) | Presence | 2 (28.6) | 5 (71.4) | 1 |
|  |  | Absence | 60 (35.5) | 109 (64.5) |  |
|  | Nephropathy (%) | Presence | 1 (25) | 3 (75) | 1 |
|  |  | Absence | 61 (35.5) | 111 (64.5) |  |
|  | Malnutrition (%) | Presence | 1 (50) | 1 (50) | 1 |
|  |  | Absence | 61 (35.1) | 113 (64.9) |  |
|  | HIV (%) | Presence | 2 (33.3) | 1 (66.7) | 0.29 |
|  |  | Absence | 60 (34.7) | 113 (65.3) |  |
|  | Miscellaneous (%) | Presence | 2 (40) | 4 (60) | 1 |
|  |  | Absence | 60 (35.3) | 110 (64.7) |  |
| Clinical presentation | Hypo or hyperthermia (%) | Presence | 35 (31.8) | 75 (68.2) | 0.17 |
|  |  | Absence | 14 (45.2) | 17 (54.8) |  |
|  | Chills (%) | Presence | 15 (34.9) | 28 (65.1) | 0.99 |
|  |  | Absence | 34 (34.7) | 64 (65.3) |  |
|  | Hypotension (%) | Presence | 27 (67.5) | 13 (32.5) | < 0.00001 |
|  |  | Absence | 22 (21.8) | 79 (78.2) |  |
|  | Diarrhoea (%) | Presence | 20 (33.9) | 39 (66.1) | 0.86 |
|  |  | Absence | 29 (35.4) | 53 (64.6) |  |
|  | Abdominal pain (%) | Presence | 15 (27.3) | 40 (72.7) | 0.14 |
|  |  | Absence | 34 (39.5) | 52 (60.5) |  |
|  | Nausea or vomiting (%) | Presence | 12 (34.3) | 23 (65.7) | 0.95 |
|  |  | Absence | 37 (34.9) | 69 (65.1) |  |
|  | Cellulitis (%) | Presence | 12 (40) | 18 (60) | 0.5 |
|  |  | Absence | 37 (33.3) | 74 (66.7) |  |
|  | Jaundice (%) | Presence | 9 (26.5) | 25 (73.5) | 0.25 |
|  |  | Absence | 40 (37.4) | 67 (62.6) |  |
|  | Ascites (%) | Presence | 8 (32) | 17 (68) | 0.76 |
|  |  | Absence | 41 (35.3) | 75 (64.7) |  |
|  | Peritonitis (%) | Presence | 5 (38.5) | 8 (61.5) | 0.77 |
|  |  | Absence | 44 (34.4) | 84 (65.6) |  |
|  | Confusion or coma (%) | Presence | 11 (55) | 9 (45) | < 0.05 |
|  |  | Absence | 38 (31.4) | 83 (68.6) |  |
|  | Meningitis and/or encephalitis (%) | Presence | 2 (40) | 3 (60) | 1 |
|  |  | Absence | 47 (34.6) | 89 (65.4) |  |
| Biology | Mean white blood cell count* (/mm^3^) (±SD, n) |  | 22091 (±47377, 26) | 12795 (±12592, 52) | 0.34 |
|  | Mean CRP rates (mg/l) (±SD, n) |  | 198 (±354, 7) | 122 (±111, 12) | 0.61 |

* Under 12 years excluded
